# Adolescent and young adult stress and coping during COVID-19: the utility of a pediatric emergency department screener

**DOI:** 10.1186/s12245-021-00359-4

**Published:** 2021-07-27

**Authors:** Ji-Ting Janet Yau, Alan L. Nager

**Affiliations:** 1 Department of Pediatrics, Children’s Hospital Los Angeles, Keck School of Medicine, University of Southern California, Los Angeles, CA USA; 2 Division of Emergency and Transport Medicine, Children’s Hospital Los Angeles, 4650 Sunset Blvd, Mailstop #113, Los Angeles, CA 90027 USA

**Keywords:** COVID-19, Mental health, Anxiety, Psychosocial stress

## Abstract

**Background:**

COVID-19 altered lives, especially adolescents and young adults who lost their emotional and social support systems and may be suffering.

**Objective:**

In response to the coronavirus pandemic, a questionnaire was created and administered to Pediatric Emergency Department (PED) patients in order to identify psychosocial stress and coping abilities.

**Methods:**

A 12-question (yes/no) quality improvement (QI) paper-based questionnaire was administered by PED providers to assess psychosocial stress and coping among patients 12 years and greater who presented to the PED at a tertiary Children’s Hospital, March-September 2020. Questions were asked/recorded to determine rates of distress and provide social work intervention, if needed. Analysis-Chi-squared, Fisher’s exact, and Mann-Whitney *U* tests.

**Results:**

Among 1261 PED patients who participated in the study, the mean age was 15.4 years (SD = 2.4), (58% female, 41.5% male, 0.6% missing data). We identified 611 patients (48.5%) who admitted to feeling scared about contracting the disease, 876 patients (69.5%) who were concerned about the health of their families, and 229 patients (18.2%) who screened positive for food insecurity. In addition, 596 patients (47.3%) felt anxiety, 333 patients (26.4%) felt depressed, and 13 patients (1%) admitted to having suicidal ideation because of COVID-19. The majority of patients, 1165 (92.4%), felt supported during the pandemic. Social work was consulted for 235 (18.6%) of patients participating.

**Conclusions:**

While patients typically present to PEDs for a somatic complaint, screening their psychosocial and emotional states may reveal underlying mental health concerns that require intervention and at times, assistance from social workers.

## Background and introduction

The COVID-19 pandemic hit the USA in early March 2020. Schools, parks, and all non-essential businesses were closed, and people were forced to stay at home in an effort to stop the spread of disease. Normal routines and activities were significantly changed. Adolescents and young adults were particularly affected, given their developing levels of emotional maturity and capacity to process and deal with their emotions and fears [1]. Children stopped physically attending school, had limitations seeing friends for social support, and some witnessed parents losing jobs. As it is known, the health of adolescents is largely affected by psychosocial factors, such as family, peers, and their social determinants of health [2]. These can all impact individuals to variable degrees, especially during a public health disaster.

The prevalence of mental health disorders in adolescents and young adults has been on the rise in the last decade [3], and this pandemic is likely to cause an even greater surge in mental health issues for this vulnerable population [4].

Although patients may present to a Pediatric Emergency Department (PED) for behavioral or psychiatric complaints, most present for medical reasons. Psychosocial stressors may be uncovered if appropriate screening is implemented. Based on a previously published screener called, the Emergency Department Distress Response Screener (ED-DRS) [5], we developed a similar Quality Improvement (QI) screener focused specifically on COVID-19. Our aim was to determine the stressors of patients and how well or ineffectively they were coping. We hypothesized that younger patients would be more distressed and have greater coping challenges during the pandemic.

## Materials and methods

In March 2020, a 12-item (yes/no) paper-based QI questionnaire was created to assess the psychosocial stress of COVID-19 on adolescents and young adults in the PED of an urban, tertiary care center with an annual volume of approximately 95,000 visits, albeit dropped to roughly 62,000 during 2020 (with COVID-19). Questions were initially created by the first author (a pediatric resident physician at the time), then revised with input from Pediatric Emergency Medicine attending physicians, psychiatrists, psychologists, and social workers at the institution until consensus was achieved. The final version of the questionnaire was sent to PED staff, with instructions on where to locate the hardcopies, how to administer confidentially, and what to do if concerning responses were elicited. Prior to this QI study, contents of the email were reviewed with all PED staff multiple times at shift change and during several unscheduled times throughout the day, evening, and night. Email and text reminders were sent to PED staff, and to pediatric residents 3-5 times a week, 2 weeks prior to study initiation. The PED nursing staff and social workers responding to PED patients were also prepared via email and in-person prior to starting data collection.

### Study population

Between March 26, 2020, and September 28, 2020, the COVID-19 Stress and Coping Questionnaire (C-19SACQ) was administered to patients 12 years and older who presented to the PED for medical complaints. Our study did not include those patients who presented for chief complaints related to mental health, such as complaints related to anxiety, depression, or those who declared themselves as having suicidal ideation. Consistent with prior research [5], exclusions pertained to patients with developmental delay or cognitive impairment, in acute crisis (trauma, severe pain, unconsciousness, medical, or surgical emergency), those who elected not to participate, those whose parents declined to leave the room, or those who communicated in a language other than English.

### Data collection

Questionnaires were administered verbally and confidentially in the privacy of the patient’s room. Identifiers (i.e., name, date of birth, or medical record number) were not recorded. The patient’s age (as a whole number) and gender were recorded.

Upon completion of the questionnaire, it was up to the clinician to use discretion whether to consult a social worker or to interpret that the patient and family were adapting sufficiently without the need for intervention. If consulted, social workers performed an assessment of the patient’s risk and provided handouts, referrals, and resources to the family for issues such anxiety, boredom, isolation, and food insecurity.

The Institutional Review Board approved all study-related activities and granted permission for data collection.

### Statistical analyses

The IBM Statistical Package for the Social Sciences (SPSS) Version 27 (Armonk, NY) was used to run analyses. Categorical variables were analyzed using Chi-squared test for associations (all expected cell counts ≥ 5), and Fisher’s exact test was used whenever an expected cell count was < 5. Post-hoc standardized residuals (*z*) were used to interpret significant associations; specifically, *z* ≥ 2.58 or −2.58 indicated salient patterns at post-hoc *p* ≤ 0.01, with negative *z* values indicating inverse patterns. Age (sole continuous variable) was analyzed using Mann-Whitney test.

## Results

A total of 1261 patients were screened; mean age was 15.4 (SD = 2.4; median = 15; range, 12-26; 9 missing). Regarding identified gender, 731 (58%) were female and 523 (41.5%) were male (7 missing).

### Primary outcome

Table 1 shows the 12-questionnaire items with “yes/no” frequencies and missing data. The majority of patients (98.4%) were aware of the pandemic, and 83.9% felt knowledgeable about COVID-19. Regarding food insecurity, 18.2% were worried about their family’s ability to obtain food. Almost half (47.3%) of medical patients surveyed admitted to experiencing anxiety, 26.4% felt depressed, and suicidal ideation was identified in 1% of patients. Social workers were consulted to perform an assessment on 18.6% of patients surveyed.

**Table 1:** The 12-questionnaire items with “yes/no” frequencies and missing data

Questions	Yes (%)	No (%)	Missing (%)
Are you aware that we have a new virus called coronavirus or COVID-19 in our community?	1241 (98.4%)	11 (0.9%)	9 (0.7%)
Do you feel emotionally supported during the coronavirus pandemic?	1165 (92.4%)	87 (6.9%)	9 (0.7%)
Have you talked to your parents about coronavirus?	1061 (84.1%)	196 (15.5%)	4 (0.3%)
Do you feel you know enough about coronavirus?	1058 (83.9%)	198 (15.7%)	5 (0.4%)
Have you talked to your friends about coronavirus?	958 (76%)	299 (23.7%)	4 (0.3%)
Are you scared or concerned about the health of your family?	876 (69.5%)	382 (30.3%)	3 (0.2%)
Are you scared or concerned about your risk of getting coronavirus?	611 (48.5%)	646 (51.2%)	4 (0.3%)
Has news about coronavirus made you feel anxious or nervous?	596 (47.3%)	662 (52.5%)	3 (0.2%)
Do you feel sad, down, or depressed because of coronavirus in the community?	333 (26.4%)	927 (73.5%)	1 (0.1%)
Are you worried about having enough food during this time of crisis?	229 (18.2%)	1026 (81.4%)	6 (0.5%)
Do you think you may be having coronavirus symptoms now?	76 (6%)	1182 (93.7%)	3 (0.2%)
Has coronavirus information made you feel distressed to the point where you want to hurt or kill yourself?	13 (1%)	1247 (98.9%)	1 (0.1%)
Social work consult	235 (18.6%)	1026 (81.4%)	0 (0%)

### Secondary outcomes

Responses to several questionnaire items were significantly associated with social work consultation (*p*’s < 0.001). More specifically, social work consultations were more likely for: patients who answered yes to being scared about their risk in contracting COVID-19 (*z* = 4.4) than those who answered no (*z* = −4.3); patients who answered yes to being scared about the health of their family (*z* = 2.8) than those who answered no (*z* = −4.3); patients who answered yes to food insecurity (*z* = 7.2) than those who answered no (*z* = −3.4); patients who answered yes to feeling anxious and nervous (*z* = 4.3) than those who answered no (*z* = −4.1); patients who answered yes to feeling depressed (*z* = 8.1) than those who answered no (*z* = −4.9); and patients who answered yes to suicidality related to COVID-19 (*z* = 3.6) than those who answered no (*z* = −0.4). Patients who answered yes to feeling emotionally supported (*z* = −1.1) were less likely to receive social work consultation compared to those who answered no (*z* = 3.9) (Tables 2 and 3).

**Table 2:** Patients who answered yes, male vs. female

	Male (%)	Female (%)	Missing (%)
Are you scared or concerned about your risk of getting coronavirus?	239 (39.1%)	369 (60.4%)	3 (0.5%)
Are you scared or concerned about the health of your family?	361 (41.2%)	511 (58.3%)	4 (0.5%)
Are you worried about having enough food during this time of crisis?	99 (43.2%)	129 (56.4%)	1 (0.4%)
Has news about coronavirus made you feel anxious or nervous?	221 (37.1%)	373 (62.6%)	2 (0.3%)
Do you feel sad, down, or depressed because of coronavirus in the community?	124 (37.2%)	208 (62.5%)	1 (0.3%)
Has coronavirus information made you feel distressed to the point where you want to hurt or kill yourself?	6 (46.2%)	7 (53.8%)	0 (0%)
Do you feel emotionally supported during the coronavirus pandemic?	473 (40.6%)	685 (58.8%)	7 (0.6%)

**Table 3:** Patients who answered yes, social work consults

	Yes (%)	No (%)
Are you scared or concerned about your risk of getting coronavirus?	161 (26.4%)	450 (73.6%)
Are you scared or concerned about the health of your family?	200 (22.8%)	676 (77.2%)
Are you worried about having enough food during this time of crisis?	89 (38.9%)	140 (61.1%)
Has news about coronavirus made you feel anxious or nervous?	157 (26.3%)	439 (73.7%)
Do you feel sad, down, or depressed because of coronavirus in the community?	126 (37.8%)	207 (62.2%)
Has coronavirus information made you feel distressed to the point where you want to hurt or kill yourself?	8 (61.5%)	5 (38.5%)
Do you feel emotionally supported during the coronavirus pandemic?	201 (17.3%)	964 (82.7%)

No significant age or gender differences were found between patients who responded yes or no to individual questions. There were also no significant age or gender differences between patients who did or did not receive a social work consultation.

## Discussion

Stress and adversity early in life may lead to overall poor health outcomes later on [6]. Physiologically speaking, the pediatric and adolescent populations have immature endocrine systems, nervous systems, and hypothalamic-pituitary-adrenal axes, which are necessary to help deal with physical and psychological stress [7]. Given the uptick in mental health issues facing adolescents and young adults prior to the pandemic, we postulated that COVID-19 would exacerbate and worsen mental health for our patients.

While many mental health screeners exist, our screener was easy to use and implement quickly because our institution already had an established mental and behavioral health screener in place (ED-DRS) [5]. We used this as the framework for questionnaire content, and tailored the questions to COVID-19. Our PED providers were already accustomed to using a mental health screener. Therefore, it was quickly accepted as a time-efficient screening tool, taking 2-5 min to complete.

Our findings are consistent with existing literature, that is, evidence of patient anxiety, depression, and even suicidality. According to our established ED-DRS screener [5], 30.4% of patients screened positive for anxiety at baseline already, and our C-19SACQ illustrated the increased prevalence of anxiety with 47.3% of patients screening positive for anxiety related to the pandemic. Additionally, Table 4 illustrates the increased prevalence of mental health disorders in our PED during the COVID-19 pandemic in 2020 as compared to 2019. Patients also had concerns about financial security and food insecurity. While the long-term downstream effects of COVID-19 on mental health in the pediatric population are still unknown, there is likely to be an effect on their mental or behavior development from the strain of living through this pandemic [7].

**Table 4:** Patient data regarding mental health conditions

	2019 (census 99,613)	2020 (census 62,454)
Patients who presented to the PED for anxiety	654 (0.66%)	716 (1.15%)
Patients who presented to the PED for depression/mood disorder	276 (0.28%)	261 (0.42%)
Patients who presented to the PED for suicidal ideation/suicide attempt	185 (0.19%)	199 (0.32%)

The majority of those who suffer from mental and psychiatric illnesses identify as female, compared to their male counterparts [8], and gender plays a role in how a person deals with stress [9]. Interestingly, this was not illustrated in our findings. Although the majority of those who were distressed and answered yes to many of our screener questions were female, gender was not a statistically significant finding. However, males tend to be less inclined to seek care or admit to psychological issues [9], a possibility in our study as well.

Of patients admitting to having difficulties coping during the pandemic, a substantial number of them were seen and evaluated by social workers; most notably, patients who screened positive for food insecurity, depression, and suicidality. Given the fact that our study was a QI initiative, our ED dedicated social workers were given the freedom to do what they felt was in the best interest of patient care, which was to assess risk, evaluate understanding, provide counseling and to provide resources and referrals either within the hospital or to outside resources.

Interestingly, age did not play a role in whether a patient responded yes or no to screening questions and age was not salient in whether or not a social work consultation occurred. We thought that the younger the patient, the more likely they would be distressed by COVID-19 and would thus, require social work assessment and intervention. We had intuitively believed younger patients would be more frightened, understand less, feel more isolated from their peers, and have immature coping abilities in a time of crisis. Our questionnaire data clearly do not depict this, perhaps suggesting some protective effect or support by their caregivers.

In a recent study performed at Yale New Haven Children’s Hospital PED, investigators tracked the frequency of patients presenting with mental health issues [10], and found a 60% reduction in visits related to mental health issues. This could be due to many reasons, including a lack of universal screening for mental and psychosocial issues at their institution. In another study performed at a tertiary care children’s hospital in Portland, Oregon, researchers found a sharp decline in pediatric mental health visits to their PED [11]. This was largely attributed to the stay-at-home order that was enacted just a week prior to their data collection. In contrast, our PED experienced an increase in patients presenting for mental health issues, as illustrated in Table 4.

Based on ED-DRS data [5] previously published, 30.4% of patients 12 years or older screened positive for anxiety at our institution pre-COVID-19. In our current study, however, 47.3% of patients admitted to feeling anxious during this pandemic. This is likely attributed to the immense uncertainty throughout the COVID-19 pandemic, such as when children will return to school, when businesses will reopen, when parents will be gainfully employed, when families will be able to gather for celebrations and religious gatherings, and when travel and entertainment will resume regular operations. Other acute stressors include, loss of routine, increased isolation, adjustment to distanced learning, abuse, grief, and difficulty accessing therapy. Living under such ambiguous and ill-defined conditions is certainly contributing to the increase in anxiety, as shown by our questionnaire.

In review of the literature, we have not seen other pediatric or general Emergency Departments screen for COVID-19-related stress and its effects on the mental, emotional, and behavioral health of presenting patients, despite providers and clinicians acknowledging its pervasive presence. Emergency Departments serve as a safety net for patients of all ages, and mental health screening should be incorporated into medical screening in order to recognize, diagnose, and provide treatment for patients early on. Given limited access to outpatient mental health services, it is even more crucial for PEDs to screen for and help patients presenting with mental health concerns [10].

Based on the breakdown of payer status for families cared for in our PED during COVID-19, 71% were government/MediCal HMO (variations on Medicaid), 26% were commercial, and 3% were deemed self-pay. It is possible that payer status was a contributor to patients and their outlook, emotional reactions, or challenges, such as food insecurity. Although it was not our intention to evaluate the relationship of emotional difficulties to finances specifically, it is possible that the financial strains of being on a welfare insurance program contributed or added to their emotional reactions and coping insecurities.

Another issue, albeit difficult to interpret, is whether patients seeking care during COVID-19 in our PED were more stressed by physically being in the PED, rather than those who may be sick, but chose to stay home. Although this is theoretically possible, our study did not study this issue, and therefore, our interpretations are limited to the subject population as depicted and analyzed.

### Limitations

Our questionnaire was created in “yes/no” format instead of Likert or qualitative formats, which limited our descriptive findings. The questionnaire was administered verbally by a provider, rather than through self-report without clinician interaction. Patients may have felt uncomfortable being honest and sharing their responses with providers due to the long-standing stigma associated with mental health conditions. Also, some providers may have proceeded to administer the questionnaire in the presence of parents who declined to temporarily exit the room. Furthermore, our questionnaire served as a screening tool, not a diagnostic tool. Lastly, it is important to note that our screener was implemented in a single PED at an inner city, tertiary children’s hospital, and therefore, our results may not be generalizable to other PEDs, populations, or settings.

## Conclusion

The mental health of the adolescent and young adult population may be at greater risk during the COVID-19 pandemic. PEDs are in a unique position to identify vulnerable patients who may have troubling emotions and difficulty coping by utilizing a short COVID-19 screener. Addressing their needs and offering mental health support constitute a worthy and valuable initiative.

## Data Availability

The datasets used and/or analyzed during the current study are available from the corresponding author on reasonable request.
